# Intraoperative single-dose methadone significantly affects postoperative morphine consumption in older patients with a hip fracture: the MetaHip randomized controlled trial

**DOI:** 10.2340/17453674.2025.44754

**Published:** 2026-04-16

**Authors:** Kevin H NYGAARD, Nikolaj H SCHMIDT, Lasse ERIKSEN, Sofie R PETERSEN, Thomas STRØM, Kirsten SPECHT, Jesper O SCHØNNEMANN

**Affiliations:** 1 Department of Orthopedics, University Hospital of Southern Denmark, Aabenraa; 2 Department of Clinical Research, University Hospital of Southern Denmark, Aabenraa; 3 Department of Anesthesia and Intensive Care, Odense University Hospital, University of Southern Denmark; 4 Department of Regional Health Research, University of Southern Denmark, Denmark

## Abstract

**Background and purpose:**

Effective pain management in older patients with a hip fracture is critical for postoperative recovery. Our primary objective was to compare intraoperative methadone with placebo on postoperative morphine consumption over 72 hours.

**Methods:**

Patients aged ≥ 60 years with hip fractures were randomized to receive methadone (0.10 mg/kg) or placebo intraoperatively. The primary outcome was postoperative morphine consumption measured in 24-hour intervals over 72 hours. Secondary outcomes included pain scores, time to mobilization, and discharge. Harms were assessed as adverse and serious adverse events.

**Results:**

129 patients were included. The primary endpoint analysis demonstrated that postoperative morphine consumption over 72 hours differed significantly between groups (likelihood-ratio test, P = 0.02). Model-based estimates suggested lower morphine consumption in the methadone group at 0–24 hours (least square mean [LSM] 7.1 [SE 1.2] vs placebo 10.1 [SE 1.7] mg) and at 24–48 hours (4.1 [SE 0.8] vs placebo 5.3 [SE 0.9] mg). At 48–72 hours, the model suggested lower morphine consumption in the placebo group (3.2 [SE 0.6] vs methadone 4.6 [SE 0.9] mg). Secondary outcomes were similar between groups, except that time to hospital discharge was longer in the methadone group (LSM 5.6 vs 4.5 days; mean difference –1.3 days, 95% confidence interval –2.3 to –o.4; P < 0.01). Harms appeared comparable, although the low event rate precluded formal analysis.

**Conclusion:**

A single intraoperative dose of methadone significantly alters postoperative morphine consumption over 72 hours after hip fracture surgery, without major safety concerns.

Older patients with a hip fracture are particularly vulnerable to side effects from analgesics, making effective and safe pain management essential [1-3]. Methadone, a long-acting opioid, has unique pharmacological properties, including N-methyl-D-aspartate receptor antagonism and inhibition of serotonin and noradrenaline reuptake [4,5]. These characteristics may enhance postoperative analgesia and reduce the need for repeated short-acting opioids [4].

Although methadone has demonstrated efficacy in reducing postoperative morphine use in younger adults undergoing elective surgery [6-14], its role in older adults undergoing acute fracture surgery remains uncertain. Clinical adoption has been limited, partly due to concerns over respiratory depression, inter-individual variability, and a lack of data from older or acutely admitted populations [4,5,15,16]. To our knowledge, methadone has never been tested in a randomized trial on older patients with a hip fracture in an acute setting. Shifting opioid administration into a controlled and closely monitored intraoperative setting may enhance consistency and reduce reliance on repeated, patient-driven dosing. This approach aligns with recent recommendations to minimize postoperative opioid titration [17]. It may also help cognitively impaired patients, who often receive inadequate analgesia, by providing consistent baseline pain relief through intraoperative dosing [18].

Our trial aimed to evaluate whether a single, standardized intraoperative dose of methadone could reduce the need for postoperative, patient-driven morphine use in older patients with a hip fracture [17,18].The trial also assessed pain scores, functional outcomes, and safety, including adverse and serious adverse events as defined by Good Clinical Practice (GCP) guidelines.

## Hypothesis

The null hypothesis was that postoperative opioid consumption during the first 72 hours did not differ between the methadone and placebo groups.

## Methods

### Trial design

We performed a double-blind randomized controlled trial. Participants were randomized in a 1:1 ratio, stratified by sex and anesthesia type. The trial was conducted in strict adherence to the published protocol [19] and the details outlined in the public registration on ClinicalTrials.gov (ID NCT06086171). The study is reported according to Hopewell et al., CONSORT 2025 Statement: updated guideline for reporting randomized trials [20].

### Participants

Details on the specific inclusion and exclusion criteria are available in the protocol [19]. Briefly, patients aged ≥ 60 years with an acute hip fracture confirmed by radiography were included, provided that they were able to ask for additional analgesics postoperatively. Patients with certain health conditions or concurrent treatments contraindicating methadone use, severe cognitive impairment, or opioid addiction were excluded.

### Setting

The trial was conducted at the University Hospital of Southern Denmark in Aabenraa, Denmark. Patients were included in the emergency department (ED). The orthopedic department treats approximately 30 patients with a hip fracture per month, reflecting a typical case volume for similar regional hospitals in Northern Europe. After surgery, patients followed a standard regimen and were observed in the post-anesthesia care unit (PACU). Patients were discharged to the orthopedic ward following standard criteria [19]. Apart from the ED nurses who prepared the trial drug, all personnel involved in patient care were blinded to the intervention.

### Interventions

Patients were randomized to receive either intravenous methadone or placebo (saline) as a single dose of 0.10 mg/kg, administered at anesthetic induction no later than 10 minutes before the first incision by a nurse anesthetist blinded to the intervention. The methadone dose was based on findings from a prior dose-adjustment study in the same population, which identified 0.10 mg/kg as the maximal tolerable dose considering the risk of respiratory depression [21]. Total bodyweight was used for dose calculation because this value is routinely available in the ED, and methadone distributes between lipophilic and hydrophilic compartments. Both groups also received a preoperative peripheral nerve block, rescue morphine, and paracetamol as part of standard care. Unblinded registered nurses (RNs) prepared the trial drugs but were not involved in patient care to maintain blinding. Further details are provided in the published protocol [19].

### Primary outcome

The primary in-hospital outcome was postoperative opioid consumption during the first 72 hours after surgery, measured in 3 consecutive 24-hour intervals (0–24, 24–48, and 48–72 hours). All opioids administered during this period were included, regardless of formulation or indication. This included both scheduled (short- or long-acting) and rescue (short-acting) opioids. Opioid doses were converted to oral morphine milligram equivalents (MME) to ensure standardized reporting across agents and routes of administration.

Postoperative opioid consumption was selected as a proxy for pain intensity based on the rationale that patients request or receive supplementary analgesia to achieve adequate pain relief. This outcome is widely used in analgesic trials and supported by previous literature [7-9,22-26].

### Co-primary outcome (3-month follow-up)

In accordance with the ClinicalTrials.gov registration (NCT06086171), opioid use at 3 months after surgery was specified as a co-primary outcome. This outcome was included to assess longer-term opioid exposure and to ensure that a single intraoperative dose of methadone did not increase the risk of persistent opioid use following hip fracture surgery.

### Primary endpoint

The primary endpoint was the overall treatment effect of methadone vs placebo on postoperative opioid consumption during the first 72 hours, evaluated using a likelihood-ratio test comparing linear mixed-effects models with and without the treatment group variable, as prespecified in the published trial protocol [19].

As this was the first trial of intraoperative methadone in a frail hip fracture population, the effect profile was uncertain with respect to timing, duration, and tolerability. Given the large inter-individual variability in postoperative pain and opioid requirements, a design focused on single time points would have required a substantially larger sample size. The trial was therefore designed to assess the overall treatment effect over 72 hours, thereby enabling an ethically acceptable first assessment of a potential treatment signal.

### Secondary outcomes and endpoints

Secondary outcomes included postoperative pain assessment (verbal rating scale), time to standing, mobility measured by the Cumulated Ambulation Score (CAS), postoperative nausea and vomiting (PONV), time to discharge, need for antidote administration, delirium assessed with the Confusion Assessment Method (CAM), and constipation. PONV, antidote use, delirium, and constipation were recorded as binary outcomes (yes/no). All outcomes were assessed once daily during the first 3 postoperative days by an RN using a standardized form, as detailed in the protocol article [19].

### Harms

Safety outcomes were assessed in accordance with Good Clinical Practice (GCP) guidelines and the independent monitoring plan. Serious adverse events (SAEs) were monitored for a duration equivalent to 5 times the half-life of methadone (~12 days). The following 5 SAE categories were predefined based on GCP recommendations:

1. Readmission or prolonged admission.
2. Life-threatening events.
3. Death.
4. Significant disability or incapacity for work.
5. Other significant medical events.

In addition, general adverse events (AEs) were recorded throughout the hospital stay. These safety outcomes were not part of the pre-specified secondary endpoints but were collected to evaluate potential harms and overall tolerability of methadone.

### Changes to trial outcomes

No changes were made to the pre-specified outcomes or endpoints after the trial commenced. This manuscript reports all pre-specified outcomes as outlined in the trial protocol [19].

### Sample size

The sample size for this RCT was predefined in the trial protocol and calculated prior to trial commencement, based on a power calculation for the originally planned Poisson regression model with clustered standard errors [19]. Intra-personal variance was estimated at 18, and previous literature reported a mean daily morphine-equivalent dose of 2.6 mg with a standard deviation (SD) of 4.6 [18]. We predefined a one-third reduction in morphine use as clinically meaningful, as specified in the trial protocol [19]. This threshold is consistent with previous randomized trials, systematic reviews, and meta-analyses of intraoperative methadone, which have typically reported relative reductions in postoperative opioid consumption of approximately 30–40% and interpreted these as clinically meaningful [6-8,12,27]. The required sample size was estimated at 65 patients per group (130 total), with a 2-sided significance level of 0.05 and an anticipated power of 88% [19].

No interim analyses were planned for this trial, and no formal stopping guidelines were established. However, the trial was designed to allow early termination if there were concerns regarding patient safety or a negative impact on the course of admission attributable to the investigational drug.

### Randomization

#### Sequence generation

Participants were randomized 1:1 to receive either methadone or placebo using block randomization (random block sizes of 4 and 6), stratified by sex and anesthesia type (general or spinal), to ensure balanced allocation across key baseline characteristics. Stratification by sex was included because the population was expected to be predominantly female, as is typical for patients with a hip fracture. The randomization sequence was generated via Research Electronic Data Capture (REDCap; https://project-redcap.org/), hosted by the Open Patient data Explorative Network at Odense University Hospital, to ensure balanced group sizes across strata.

#### Allocation concealment mechanism

Pre-packaged ampoules were prepared by Glostrup Pharmacy according to the randomization sequence. Unblinded RNs in the ED accessed these ampoules and prepared indistinguishable syringes containing either methadone or saline, ensuring blinding of all patient care personnel, including anesthesiology and ward staff, as well as the patients themselves.

#### Implementation

The orthopedic physician overseeing patient inclusion generated the randomization sequence in REDCap. Unblinded RNs prepared and labeled the investigational syringes with a random treatment number before placing them near the patient, ensuring no further involvement in trial procedures. The investigational drug was administered intravenously at anesthesia induction, no later than 10 minutes before the first incision (“knife-to-skin”), minimizing protocol deviations.

### Blinding

This was a double-blind trial. Blinding was maintained for all participants and personnel after intervention assignment, including anesthesiology staff, orthopedic doctors, ward staff, outcome assessors, and the study investigator. The intervention appeared identical in volume, color, and labeling for both groups. Unblinding occurred only after the final trial audit and completion of all statistical analyses. The study investigator was the only team member unblinded at the patient level, having received the randomization list. Supervisors and co-authors remained blinded at the patient level but participated in manuscript preparation after group-level unblinding. Participants will be informed of their assigned treatment and provided with a lay summary once results have been published in a peer-reviewed journal.

### Statistics

Baseline characteristics were summarized using counts and proportions for categorical variables and medians with interquartile ranges (IQRs) for continuous variables, because these were not normally distributed, as indicated by visual inspection and distribution plots.

To account for the multiplicative nature of the treatment effect, we used a log-linear mixed-effects regression model to analyze morphine consumption across 3 consecutive 24-hour intervals (0–24, 24–48, and 48–72 hours). Because the treatment effect was expected to vary over time, morphine consumption was analyzed as repeated measurements rather than a single cumulative total, allowing the time course of the treatment effect to be assessed. The likelihood-ratio test evaluated whether morphine consumption differed between groups over the entire 72-hour postoperative period. This approach was prespecified in the protocol due to uncertainty regarding methadone’s duration of action and because repeated-measures modelling makes more efficient use of available data (19). Although a mixed-effects Poisson regression was prespecified, diagnostic evaluation revealed overdispersion, indicating a violation of the mean–variance assumption. A log-linear mixed-effects model provided a better fit. The model included time as an interaction term and random intercepts at the patient level. Model fit was evaluated graphically and deemed adequate based on the derived residual plots. Results are presented as least squares means (LSMs) with standard errors (SE), along with model-based absolute differences and ratios (placebo vs methadone).

Time to first mobilization and time to hospital discharge were analyzed using both Cox proportional hazards regression and generalized linear models.

Secondary continuous outcomes were analyzed using mixed-effects linear regression models. Single-timepoint binary outcomes were analyzed using chi-square or Fisher’s exact test, as appropriate. The Bonferroni method was used to adjust for multiple comparisons of secondary outcomes, maintaining a shared family-wise error rate. All analyses followed the pre-specified statistical approach detailed in the published study protocol [19]. Analyses were conducted according to the intention-to-treat principle. All statistical analyses were performed using Stata version 18 (StataCorp, College Station, TX, USA).

### Subgroup analyses

Pre-specified subgroup analyses by anesthesia type were deemed unnecessary, as stratified randomization ensured balanced allocation and helped minimize confounding. While some effect modification cannot be ruled out, the trial was designed to evaluate methadone as part of a multimodal analgesic approach intended to be broadly applicable to patients with a hip fracture, regardless of anesthesia technique. Thus, the primary interest was the overall treatment effect rather than stratum-specific effect modification. Instead, we explored fracture type (collum femoris, intertrochanteric, subtrochanteric) as an interaction term in the mixed-effects model. No additional subgroup analyses were pre-specified or performed.

### Ethics, data sharing plan, funding, use of AI, and disclosures

The trial was registered on ClinicalTrials.gov (ID: NCT06086171) on October 4, 2023, and in the EU Clinical Trials Information System (EU-CTIS, ID: 2023–506252-24–00) with a decision date of October 2, 2023. This study’s Universal Trial Number (UTN) is U1111-1294–6125. The study protocol was submitted to Trials on May 29, 2024, and published on December 20, 2024 [19] and is also available through the Clinical Trials Information System (CTIS): https://euclinicaltrials.eu/search-for-clinical-trials/?lang=en&EUCT=2023-506252-24-00. These resources provide comprehensive details concerning the study design, methods, and planned analyses. The Danish Medicines Agency and the National Committee on Health Research Ethics approved the trial simultaneously via CTIS, with a decision date of October 2, 2023.

Anonymized data from this study can be shared with individuals upon reasonable request for research purposes. Each request must include a clear rationale and will be evaluated on a case-by-case basis.

Funding was provided by grants from The A.P. Møller and Chastine Mc-Kinney Møller Foundation (grant number L-2022–00365), the Knud and Edith Eriksens Memorial Foundation (grant number 62786–2023), the University Hospital of Southern Denmark (grant number 22/25,256), and the Region of Southern Denmark (grant number 2/26,251). Funders did not influence data analysis, interpretation, or publication.

The authors acknowledge the use of generative AI tools, including ChatGPT-4.0 and Grammarly Premium, for language editing to enhance grammar, readability, and overall manuscript flow. The authors take full responsibility for the accuracy and integrity of the manuscript’s content.

All authors declare no conflicts of interest. The funders and study setting had no role in the study’s design, conduct, data analysis, interpretation of results, or decision to publish. Complete disclosure of interest forms according to ICMJE are available on the article page, doi: 10.2340/17453674.2025.44754

## Results

### Participant flow and recruitment

**Figure 1:**
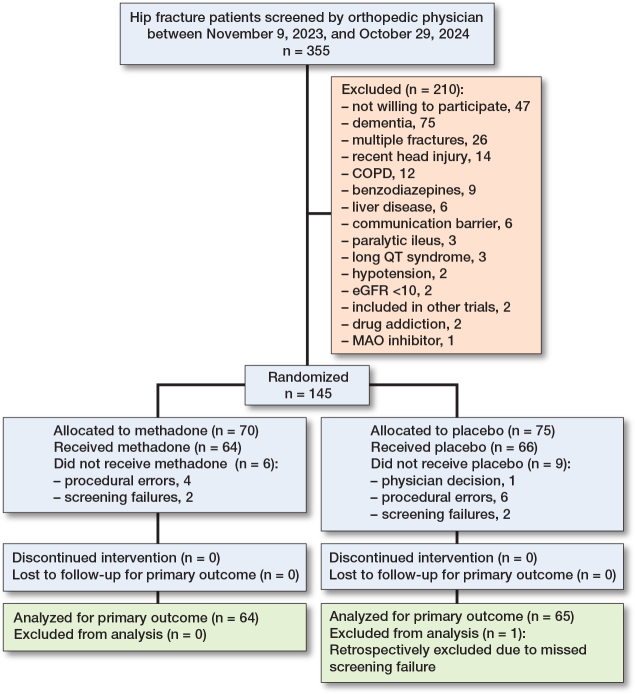
Patient flow diagram showing the enrollment, allocation, exclusions, follow-up, and analysis of patients. COPD = chronic obstructive pulmonary disease; communication barrier = unable to understand Danish or unable to request supplementary analgesics; eGRF = estimated glomerular filtration rate; MOA = monoamine oxidase. A full list of exclusion criteria is provided in the published protocol [19].

Between November 9, 2023, and October 29, 2024, 355 patients with a hip fracture were screened. Of these, 145 were randomized, and 129 were included in the intention-to-treat population and primary analysis after exclusions due to procedural errors, screening failures, and clinical decisions. These included 64 in the methadone group and 65 in the placebo group (Figure 1).

### Intention-to-treat population baseline data

Baseline characteristics showed that the median age was 82.0 years (IQR 75.0–87.0), and 83 participants (64%) were female (Table 1). The most common comorbidities were uncomplicated hypertension (76; 59%), cardiac arrhythmias (43; 33%), and mixed neurological disorders (24; 19%). An ASA score of 3 was recorded in 71 participants (55%). Baseline characteristics were similar between groups.

**Table 1:** Baseline characteristics of participants. Categorical variables are presented as counts with percentages of the group (column %), and continuous variables as medians with IQRs.

Variables	Methadone n = 64	Placebo n = 65	Combined n = 129
Hip fracture type			
Collum femoris fracture (DS720)	35	33	68 (53)
Intertrochanteric fracture (DS721)	24	29	53 (41)
Subtrochanteric fracture (DS722)	5	3	8 (6.2)
Anesthesia			
Spinal	31	34	65 (50)
General	33	31	64 (50)
Demographic data			
Age, median (IQR)	83 (73–87)	82 (76–88)	82 (75–87)
Female sex	39	44	83 (64)
Male sex	25	21	46 (36)
ASA classification			
1	4	4	8 (6.2)
2	24	23	47 (36)
3	34	37	71 (55)
4	2	1	3 (2.3)
Lifestyle			
Bodyweight, kg, median (IQR)	70 (60–78)	67 (56–77)	68 (60–77)
Body mass index, median (IQR)	24 (22–27)	23 (21–26)	24 (22–26)
Smoking	9	14	23 (18)
Alcohol, units per week			
0	23	23	46 (36)
< 10	37	38	75 (58)
> 10	4	4	8 (6.2)
Chronic use of opioids	4	2	6 (4.7)
Comorbidities			
Congestive and chronic heart failure	3	1	4 (3.1)
Cardiac arrhythmias	23	20	43 (33)
Valvular disease	7	3	10 (7.8)
Pulmonary circulation disorders	1	0	1 (0.8)
Peripheral vascular disorders	3	1	4 (3.1)
Hypertension, uncomplicated	42	34	76 (59)
Hypertension, complicated	0	0	0 (0.0)
Paralysis	2	1	3 (2.3)
Mixed neurological disorders	11	13	24 (19)
Chronic pulmonary disease	5	5	10 (7.8)
Diabetes, uncomplicated	10	6	16 (12)
Diabetes, complicated	3	0	3 (2.3)
Hypothyroidism	3	2	5 (3.9)
Chronic kidney disease	8	2	10 (7.8)
Liver disease	0	0	0 (0.0)
Malignancy	5	6	11 (8.5)
Osteoporosis	3	11	14 (11)

ASA = American Society of Anesthesiologists.

### Primary outcomes and endpoint

#### Primary outcome: postoperative morphine consumption

Median morphine consumption was lower in the methadone group during the first 2 intervals (0–24 and 24–48 hours) and higher during the last interval (48–72 hours) (Table 2).

**Table 2:** Descriptive primary outcome data with distribution summary for the postoperative morphine consumption. Values are median mg with interquartile range (IQR)

Time interval	Methadone (n = 64)	Placebo (n = 65)
0–24 hours	10 (0.0–20)	13 (10–20)
24–48 hours	2.5 (0.0–20)	10 (0.0–20)
48–72 hours	6.3 (0.0–10)	0.0 (0.0–10)

#### Primary endpoint

**Figure 2:**
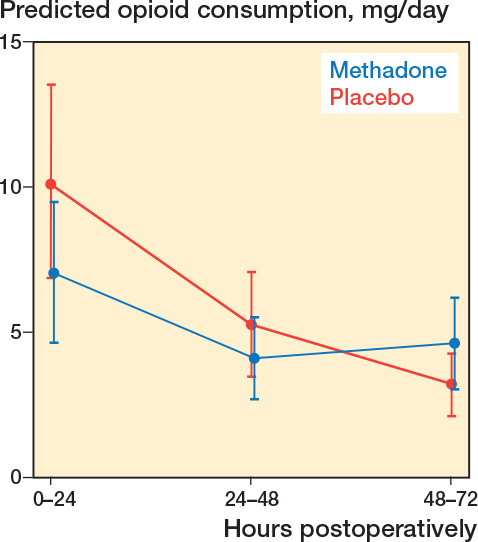
The margins plot shows morphine consumption over time in the 2 groups and illustrates the difference underlying the primary endpoint which was a likelihood-ratio test (LRT) comparing nested mixed-effects log-linear models with and without the treatment group variable. The test demonstrated a statistically significant between-group difference (P = 0.02).

A global likelihood-ratio test showed that postoperative morphine consumption differed significantly between groups across the 72-hour period in favor of methadone (P = 0.02) (Figure 2). Model-based estimates showed that during 0–24 hours morphine consumption was 7.1 mg (SE 1.2) in the methadone group and 10.1 mg (SE 1.7) in the placebo group. The model-based absolute difference was 3.0 mg (95% confidence interval [CI] –1.1 to 7.2). During 24–48 hours, the difference was 1.2 mg (CI –1.1 to 3.5). During 48–72 hours, morphine consumption was 1.4 mg lower in the placebo group (CI –3.3 to 0.5). The model-based ratios (placebo vs methadone) were 1.43 (CI 0.88–3.31) for 0–24 hours, 1.29 (CI 0.73–1.84) for 24–48 hours, and 0.70 (CI 0.43–1.12) for 48–72 hours (Table 3).

**Table 3:** Descriptive primary outcome data with morphine consumption group estimates (in mg). Values are least square mean (LSM) with standard error (SE) or absolute difference and ratio with 95% confidence intervals (CI)

Time interval	Methadone (n = 64) LSM (SE)	Placebo (n = 65) LSM (SE)	Absolute difference: Placebo – methadone (CI)	Ratio: Placebo/methadone (CI)
0–24 hours	7.1 (1.2)	10.1 (1.7)	3.0 (–1.1 to 7.2)	1.43 (0.88–3.31)
24–48 hours	4.1 (0.7)	5.3 (0.9)	1.2 (–1.1 to 3.5)	1.29 (0.73–1.84)
48–72 hours	4.6 (0.8)	3.2 (0.6)	–1.4 (–3.3 to 0.5)	0.70 (0.43–1.12)

#### Co-primary outcome: morphine consumption 3 months postoperatively

At 3 months after surgery, continued opioid use was reported by 6 patients (7.2%): 3/64 in the methadone group and 3/65 in the placebo group (Table 4).

**Table 4:** Co-primary outcome: continued opioid use at 3 months after surgery among patients with available 3-month follow-up data (n = 83). Values are presented as counts with percentages, risk difference, and odds ratio with 95% confidence intervals (CI)

Methadone (n = 42)		Placebo (n = 41)		Risk difference: Placebo – methadone (CI)	Odds ratio (CI)	P
n	% (CI of %)	n	% (CI of %)			
3	7.1 (1.5–19.5)	3	7.3 (1.5–20)	0.2 (–11 to 11)	1.0 (0.2–5.6)	1

Odds ratio and P value are derived from Fisher’s exact test.

### Secondary outcomes

Descriptive distribution summaries of secondary outcomes are presented in Tables 5 and 6, and model-based endpoint estimates are presented in Tables 7 and 8.

Time to hospital discharge differed between groups (Table 7). The model-based LSM length of stay was 5.6 days (SE 0.4) in the methadone group and 4.5 days (SE 0.2) in the placebo group. The mean difference was –1.3 days (CI –2.2 to –0.4; P < 0.01).

All other secondary outcomes were similar between groups (Tables 5–8). Antidote administration occurred only once, precluding further statistical analysis.

**Table 5:** Descriptive secondary outcome data. Repeated measurements (CAS and pain scores) are summarized as medians with interquartile ranges (IQRs) for each 24-hour interval. Time-to-event outcomes are presented as medians with IQR.

Item	Methadone (n = 64)	Placebo (n = 65)
Cumulated ambulation score		
0–24 hours	3.0 (2.0–3.0)	3.0 (2.0–3.0)
24–48 hours	3.0 (2.0–5.0)	3.0 (2.0–5.0)
48–72 hours	3.0 (2.0–5.0)	3.0 (2.0–6.0)
Pain score at rest		
0–24 hours	0.0 (0.0–1.0)	0.5 (0.0–1.0)
24–48 hours	0.0 (0.0–1.0)	0.0 (0.0–1.0)
48–72 hours	0.0 (0.0–1.0)	0.0 (0.0–1.0)
Pain score when mobilized		
0–24 hours	2.0 (1.0–2.0)	2.0 (1.0–2.0)
24–48 hours	2.0 (1.0–2.0)	2.0 (1.0–2.0)
48–72 hours	1.0 (1.0–2.0)	1.0 (1.0–2.0)
Time to standing, hours	14.9 (10.5–18.1)	11.9 (8.8–18.0)
Time to discharge, days	4.8 (3.8–6.9)	4.0 (3.0–5.6)

**Table 6:** Descriptive secondary outcome data. Values are count with percentages and 95% confidence intervals (CIs)

Item	Methadone (n = 64)		Placebo (n = 65)	
	n	% (CI of %)	n	% (CI of %)
Constipation	15	23 (14–36)	13	20 (11–32)
Delirium	5	7.8 (2.6–17)	2	3.1 (0.4–11)
PONV	16	25 (15–37)	14	22 (12–34)
Antidote	Not applicable	Not applicable		

PONV = postoperative nausea and vomiting.

**Table 7:** Secondary endpoints with binary outcomes presented as risk differences and odds ratios with corresponding 95% confidence intervals (CI) and P values

Item	Risk difference (CI), %	Odds ratio (CI)	P value
Constipation	3.4 (–11 to 18)	1.2 (0.5–2.9)	0.7 **^a^**
Delirium	4.7 (–3.1 to 13)	2.5 (0.4–27.4)	0.4 **^b^**
PONV	3.5 (–11 to 18)	1.2 (0.5–2.8)	0.7 **^a^**
Antidote **^c^**	Not applicable		

a Chi-square test.

b Fisher’s exact test.

c Not analyzed separately because very low event count precluded meaningful analysis and posed a risk of patient identifiability.

PONV = postoperative nausea and vomiting.

**Table 8:** Secondary endpoints analyzed using prespecified generalized linear models (GLM). Repeated measurements and time-to-event outcomes are presented as model-based least squares mean (LSM) with standard error (SE) and difference with 95% confidence interval (CI) and P values derived from GLM

Item	Methadone (n = 64) LSM (SE)	Placebo (n = 65) LSM (SE)	Difference: Placebo – methadone (CI)	P value
Cumulated ambulation score				0.6
0–24 hours	2.9 (0.2)	2.8 (0.2)	–0.1 (–0.7 to 0.5)	
24–48 hours	3.2 (0.2)	3.4 (0.2)	0.2 (–0.4 to 0.7)	
48–72 hours	3.6 (0.2)	3.8 (0.2)	0.2 (–0.4 to 0.8)	
Pain score at rest				0.4
0–24 hours	0.6 (0.1)	0.7 (0.1)	0.2 (–0.1 to 0.4)	
24–48 hours	0.4 (0.1)	0.6 (0.1)	0.3 (0.0 to 0.5)	
48–72 hours	0.4 (0.1)	0.5 (0.1)	0.1 (–0.1 to 0.4)	
Pain score when mobilized				0.8
0–24 hours	1.7 (0.1)	1.9 (0.1)	0.2 (–0.1 to 0.5)	
24–48 hours	1.5 (0.1)	1.6 (0.1)	0.1 (–0.2 to 0.4)	
48–72 hours	1.4 (0.1)	1.5 (0.1)	0.1 (–0.1 to 0.4)	
Time to mobilization, hours	17.1 (2.2)	13.2 (1.4)	–3.9 (–8.30 to 0.5)	0.1
Time to discharge, days	5.6 (0.4)	4.5 (0.2)	–1.3 (–2.3 to –0.4)	< 0.01

### Harms

36 adverse events were reported: 19 events (30%) in the methadone group and 17 events (26%) in the placebo group (Table 9). Serious adverse events occurred in 4/64 of patients in the methadone group and 8/65 in the placebo group. Readmission or prolonged hospitalization occurred in 2/64 methadone-treated patients and 8/65 placebo-treated patients. The low number of events precluded formal statistical analysis. No adverse events or serious adverse events were assessed as related to the investigational drug.

**Table 9:** Harms: absolute incidence of adverse events (AEs) and serious adverse events (SAEs) by group. Results are presented as counts and percentages with 95% confidence intervals (CIs). Several SAEs are censored due to limited observations and risk of patient identifiability. Overall, very low event count precluded meaningful analysis

Outcome	Methadone (n = 64)		Placebo (n = 65)	
	n	% (CI of %)	n	% (CI of %)
Adverse events (AE)	19	30 (19–42)	17	26 (16–39)
Serious adverse events (SAE)	4	6.3 (1.8–15)	8	12 (5.5–23)
Readmission or prolonged admission	2	3.1 (0.4–11)	8	12 (5.5–23)
Life-threatening		Censored		Censored
Death		Censored		Censored
Significant disability/incapacity for work		Censored		Censored
Other significant medical event		Censored		Censored

### 3-month follow-up

Of the 129 randomized patients, 83 (64%) completed the 3-month follow-up. 8 patients were deceased at the time of follow-up, including 3/64 in the methadone group and 5/65 in the placebo group (Table 10). At 3 months, all follow-up outcomes were similar between groups (Table 11).

**Table 10:** 3-month follow-up status and baseline characteristics for the 83 patients who completed the follow-up. Values are count unless specified

Item	Methadone (n = 64)	Placebo (n = 65)	Combined (n = 129)
Dead at 3 months	3	5	8
Complete follow-ups	42	41	83
Median age, years (IQR)	83 (75–87)	80 (74–86)	81 (74–87)
Female sex	22	28	50
ASA class			
1	0	2	2
2	19	16	35
3	23	23	46
Fracture type			
Collum femoris	23	19	42
Intertrochanteric	15	20	35
Subtrochanteric	4	2	6
Persistent adverse effects (any) **^a^**	3	6	9

a Persistent adverse effects were defined as patient-reported dizziness, drowsiness, nausea, vomiting, or constipation, assessed during the structured follow-up interviews.

**Table 11:** 3-month follow-up of secondary outcome scores. Worst-case scenarios assume zero scores for missing patients. Values are median score with interquartile ranges (IQR) and difference with 95% confidence interval (CI)

	Methadone (n = 42)	Placebo (n = 41)	Difference: Placebo – methadone (CI)	P value
New Mobility Score (NMS)	6 (4–9)	6 (6–9)	–	0.4 **^a^**
Pain score at rest	0 (0–0)	0 (0–0)	–	1
Pain score when mobilized	1 (0–2)	1 (0–2)	–	0.5 **^a^**
EQ-5D-5L Index	0.92 (0.84–0.96)	0.93 (0.84–0.97)	0.01 (–0.05 to 0.06)	0.9 **^b^**
EQ-5D-5L VAS	70 (50–80)	75 (60–80)	4.4 (–3.7 to 12.5)	0.3 **^b^**
EQ-5D-5L Index (worst case)	0.91 (0.82–0.96)	0.92 (0.81–0.97)	–0.03 (–0.16 to 0.10)	0.6 **^b^**
EQ-5D-5L VAS (worst case)	65 (50–80)	73 (50–80)	1.1 (–9.9 to 12.2)	0.8 **^b^**

a Wilcoxon rank-sum tests for non-parametric outcomes.

b Generalized linear models (GLMs).

### Subgroup analyses

Exploratory subgroup analyses by fracture type are presented in the supplementary material (Supplementary Tables 12–13). Baseline morphine consumption differed between fracture types (Supplementary Table 12). Patients with an intertrochanteric fracture had higher baseline morphine consumption and showed the largest model-based differences in postoperative morphine consumption between groups (Supplementary Table 13).

## Discussion

Our primary objective was to compare intraoperative methadone with placebo on postoperative morphine consumption over 72 hours. We found that postoperative morphine consumption differed statistically significantly with a reduction of 1 to 3 mg morphine between the methadone and placebo groups during the first 72 hours after hip fracture surgery. As this was the first randomized trial of intraoperative methadone in a frail hip fracture population, the effect profile was uncertain with respect to timing, duration, and tolerability. Consequently, the study was designed to assess an overall treatment signal across repeated postoperative intervals, rather than to provide precise effect estimates at individual time points.

Taken together, these findings support the interpretation of the MetaHip trial as a proof-of-concept study demonstrating that a single intraoperative dose of methadone can significantly alter postoperative opioid use in older patients with a hip fracture. The acceptable safety profile observed in this trial further supports the feasibility of continued investigation in this setting.

The observed between-group difference during the first 24 hours (ratio 1.43; see Table 3), corresponding to 43% higher morphine use in the placebo group, is consistent with prior studies reporting relative reductions in morphine use in the range of 25–50% [7,8,20]. However, the trial was designed to evaluate an overall group difference across the 72-hour period using a global test [30], and time-specific estimates should therefore be interpreted as exploratory.

An absolute MCID of 5 mg has been proposed in previous literature, based on arthroplasty populations with substantially higher baseline morphine consumption (median 28 mg [IQR 18–44] during 0–24 hours) [28]. In contrast, the baseline consumption in the placebo group of our trial was 10.1 mg (see Table 3). Applying a fixed absolute threshold derived from a population with nearly threefold higher baseline requirements would require a reduction exceeding 50–70% of total morphine use in our cohort, which is mathematically implausible and clinically unreasonable.

Consequently, the clinical relevance of the absolute reduction observed in this trial cannot be determined using existing absolute MCID thresholds, but should be interpreted in a relative, population-specific context.

### Strengths

This study is a pre-registered protocol [19], double-blind design, and focused on a clinically relevant, underexplored population. The trial was conducted at a single site, which may limit generalizability. However, this study’s population closely resembles the typical hip fracture demographic in Western countries in terms of age, gender distribution, and health status [1,2]. This similarity enhances the external validity of our findings, suggesting that the results may apply to broader healthcare settings, even if the exclusion of patients with severe organ dysfunction or cognitive impairment may limit some applicability to frailer patients often seen in routine clinical care.

### Limitations

This trial was powered only for the primary endpoint. Analyses of secondary outcomes and interval-specific estimates should therefore be interpreted as exploratory. Another limitation is that the morphine consumption estimate used for the power calculation (2.6 mg/day, SD 4.6) [18] was lower than the median morphine use observed in this trial. However, this reflects a mean-to-median comparison and should be interpreted with caution. The calculation also targeted a relative reduction, making variability more important than the absolute mean. The observed treatment effect and variance in our data aligned with the initial assumptions, supporting the study’s sufficiency of power.

The higher morphine use in this trial may reflect differences in clinical prescribing practices or institutional pain thresholds. The reference cohort used for the power calculation reported lower morphine use but also a higher incidence of severe pain scores [18]. In contrast, participants in this trial received more morphine but also reported lower pain scores. This could indicate a more liberal opioid-prescribing approach or a lower tolerance for postoperative pain in our setting.

Because morphine was administered only upon patient request, a possible reduction with stable pain scores can be interpreted as indicative of better analgesic efficacy. This approach is supported by similar trials where lower morphine use alongside similar pain scores has been interpreted as evidence of superior analgesia [23,25,29,30]. This may be particularly relevant for cognitively impaired patients, who often receive insufficient analgesia in patient-driven regimens. A standardized intraoperative methadone dose offers equitable baseline analgesia, independent of a patient’s ability to request analgesics. However, methadone’s analgesic benefits appeared time-limited, as morphine consumption increased in the methadone group beyond 48 hours. This reversal in morphine requirements may indicate a rebound pain phenomenon. The slightly prolonged discharge time in the methadone group may further support this interpretation, possibly reflecting challenges in later-phase pain management [19].

### Conclusion

Our randomized controlled trial demonstrates that a single intraoperative dose of methadone significantly influences postoperative morphine requirements during the first 72 hours after hip fracture surgery, but there is uncertainty regarding the clinical relevance.

*In perspective*, the role of methadone as a potential adjunct to multimodal analgesia in older patients with a hip fracture should be further evaluated before implementation in the daily routine. Future trials should focus on optimized dosing strategies, including repeated or dual-dose regimens, identification of patient subgroups most likely to benefit, and confirmation of the clinical relevance of the treatment-related changes in postoperative morphine consumption.

### Supplementary data

Supplementary Tables 12 and 13 are available on the article home page, doi: 10.2340/17453674.2025.44754

## Supplementary Material



## Notes

KHN served as the lead investigator and is the first and corresponding author. KHN made substantial contributions to the conception and design of the study, data acquisition, analysis, interpretation, and drafting of the manuscript. NHS and LE contributed significantly to data acquisition and drafting of the manuscript. SRP, as the lead biostatistician, made substantial contributions to the analysis, interpretation of data, and drafting of the manuscript. KS and TS, as co-supervisors, contributed substantially to the conception and design of the study and the interpretation of data. JOS, as the main supervisor, primary investigator, study sponsor, and head of the research group, made substantial contributions to the conception and design of the study, data acquisition, and interpretation of data.

All authors critically reviewed the manuscript, approved the final version for publication, and agreed to be accountable for all aspects of the work to ensure its integrity and accuracy. The authors affirm that their contributions adhere to the International Committee of Medical Journal Editors (ICMJE) criteria for authorship.

The authors extend their appreciation to the University Hospital of Southern Denmark for providing access to their facilities, enabling the successful conduct of this trial. They are particularly grateful to the Departments of Orthopedics, Emergency Department, and Anesthesiology and Intensive Care for their collaboration and support. Furthermore, they deeply appreciate the invaluable guidance and assistance provided by the personnel within the Department of Clinical Research. Finally, they would like to thank all individuals who contributed to the project, including the dedicated healthcare staff and others who assisted during the trial.

Handling co-editors: Taco Gosens and Robin Christensen

*Acta* thanks Nicolai Bang Foss and Barbara Maat for help with peer review of this manuscript.
