# Supplementary material for: Intraoperative single-dose methadone significantly affects postoperative morphine consumption in older patients with a hip fracture: the MetaHip randomized controlled trial
Source: Acta Orthop. 2026 Apr 16;97:255–64. doi: 10.2340/17453674.2025.44754 (PMC13093133; doi:10.2340/17453674.2025.44754)
Supplement: Supplementary file 1 [file ActaO-97-44754-s1.pdf]

Supplementary materials

Table 12. Subgroup analyses. Distribution summary for the morphine consumption (in mg) for the different fracture types. Values are median with interquartile range (IQR)

Time interval	Collum femoris (n = 68)	Intertrochanteric (n = 53)	Subtrochanteric (n = 8)
0–24 hours	10 (0.0–20)	15 (10–25)	10 (7.5–38)
24–48 hours	0.0 (0.0–10)	10 (0.0–20)	10 (5.0–23)
48–72 hours	0.0 (0.0–10)	10 (0.0–20)	15 (5.0–25)

Table 13. Results from the mixed-effects log-linear regression model with group, time, and fracture type as interaction terms and random intercepts at the patient level. Values are presented as model-based least squares mean (LSM) with standard error (SE) and difference and ratio with 95% confidence interval (CI)

Interaction terms	Methadone (n = 64) LSM (SE), mg	Placebo (n = 65) LSM (SE), mg	Absolute difference: Placebo – methadone (CI), mg	Ratio: Placebo/methadone (CI)
**Group#Time**				
0–24 hours	5.9 (1.3)	7.7 (1.8)	1.8 (–2.6 to 6.2)	1.30 (0.69–3.46)
24–48 hours	3.9 (0.9)	3.4 (0.8)	-0.5 (–2.8 to 1.8)	0.68 (0.35–2.28)
48–72 hours	3.5 (0.8)	1.8 (0.4)	–1.7 (–3.5 to 0.1)	0.40 (0.21–0.76)
**Group#Time#Fracture**				
0–24 hours#Intertrochanteric	7.8 (2.1)	13.5 (3.3)	5.7 (–2.0 to 13.5)	1.33 (0.51–4.46)
0–24 hours#Subtrochanteric	15.5 (9.2)	12.1 (9.3)	–3.5 (–29.1 to 22.2)	0.60 (0.08–5.44)
24–48 hours#Intertrochanteric	3.8 (1.0)	8.7 (2.2)	4.9 (0.2 to 9.6)	1.95 (0.73–6.21)
24–48 hours#Subtrochanteric	9.4 (5.6)	5.8 (4.5)	–3.6 (–17.7 to 10.5)	1.18 (0.15–10.1)
48–72 hours#Intertrochanteric	4.9 (1.3)	6.3 (1.6)	1.4 (–2.7 to 5.4)	1.86 (0.71–5.59)
48–72 hours#Subtrochanteric	22.2 (13.2)	2.2 (1.7)	–20.1 (–46.2 to 6.1)	0.32 (0.04–3.44)
